# The analysis on the human protein domain targets and host-like interacting motifs for the MERS-CoV and SARS-CoV/CoV-2 infers the molecular mimicry of coronavirus

**DOI:** 10.1371/journal.pone.0246901

**Published:** 2021-02-17

**Authors:** Yamelie A. Martínez, Xianwu Guo, Diana P. Portales-Pérez, Gildardo Rivera, Julio E. Castañeda-Delgado, Carlos A. García-Pérez, José A. Enciso-Moreno, Edgar E. Lara-Ramírez

**Affiliations:** 1 Unidad de Investigación Biomédica de Zacatecas, Instituto Mexicano Del Seguro Social, Zacatecas, México; 2 Laboratorio de Inmunología y Biología Celular y Molecular, Facultad de Ciencias Químicas, Universidad Autónoma de San Luis Potosí, San Luis Potosí, México; 3 Laboratorio de Biotecnología Genómica, Centro de Biotecnología Genómica, Instituto Politécnico Nacional, Reynosa, México; 4 Laboratorio de Biotecnología Farmacéutica, Centro de Biotecnología Genómica, Instituto Politécnico Nacional, Reynosa, México; 5 Cátedras-CONACYT, Unidad de Investigación Biomédica de Zacatecas, Instituto Mexicano Del Seguro Social, Zacatecas, México; 6 Information and Communication Technology Department (ICT), Complex Systems, Helmholtz Zentrum München, Neuherberg, Germany; Institute of Parasitology and Biomedicine, SPAIN

## Abstract

The MERS-CoV, SARS-CoV, and SARS-CoV-2 are highly pathogenic viruses that can cause severe pneumonic diseases in humans. Unfortunately, there is a non-available effective treatment to combat these viruses. Domain-motif interactions (DMIs) are an essential means by which viruses mimic and hijack the biological processes of host cells. To disentangle how viruses achieve this process can help to develop new rational therapies. Data mining was performed to obtain DMIs stored as regular expressions (regexp) in 3DID and ELM databases. The mined regexp information was mapped on the coronaviruses’ proteomes. Most motifs on viral protein that could interact with human proteins are shared across the coronavirus species, indicating that molecular mimicry is a common strategy for coronavirus infection. Enrichment ontology analysis for protein domains showed a shared biological process and molecular function terms related to carbon source utilization and potassium channel regulation. Some of the mapped motifs were nested on B, and T cell epitopes, suggesting that it could be as an alternative way for reverse vaccinology. The information obtained in this study could be used for further theoretic and experimental explorations on coronavirus infection mechanism and development of medicines for treatment.

## Introduction

Coronaviruses (CoV) are enveloped single-stranded, positive-sense RNA viruses, responsible very often for mild upper respiratory infections in humans. Nevertheless, remarkably pathogenic CoVs to humans have been reported. The first one appeared in 2003 in Guangdong, China, leading to an epidemic of severe acute respiratory syndrome (SARS) and this virus was named SARS-CoV [1]. In 2012, another CoV arose in Middle Eastern countries, causing pneumonic syndrome, called MERS-CoV [2]. At the end of 2019, a new CoV emerged in Wuhan, China, causing severe pneumonia [3] and was named SARS-CoV-2 due to its genomic similarity with the past SARS-CoV [4]. This is the first CoV that caused a pandemic disease termed COVID-19. These three CoVs are zoonotic, and its primary origin was traced to bats and other animals [4, 5]. We are still suffering from SARS-CoV-2. This is a serious public health concern, especially for the aged people with increased risk for complications such diabetes mellitus (DM), hypertension, and severe obesity, which cause the high morbidity-mortality rates of COVID-19 [6]. Humans infected by SARS-CoV-2 could be also asymptomatic, but they may transmit the virus [6]. Although numerous efforts are currently underway to develop drugs and vaccines to combat those viruses, there is no effective treatment available yet.

The study on molecular interactions of host-pathogen helps to find new targets for drug discovery or antigens for vaccine development. Host-pathogen relation is mainly explored through protein-protein interaction (PPI) studies. These studies can be experimentally and computationally aided [7]. The computational studies could be preliminary but quick to guide the rational selection of data for experimental confirmations. Experimental approaches have been carried out for SARS-CoV, MERS-CoV [8, 9], and recently for SARS-CoV-2 [10]. A detailed literature mining that surveys experimental and predicted PPIs for several coronaviruses, including the viruses studied herein, was recently published [11]. Also, several computation-aided researches focused on predicting PPI of host and SARS-CoV-2 [7, 12, 13]. Such predictions provided valuable information to help the rational design of treatments against these viral infections.

However, the analysis of domain-motif interaction (DMI) has paid less attention to those CoVs. Domains in proteins are the functional units involved in the signaling networks within a cell [14]. Its length is up to 200 amino acids, and its folding patterns are independent of the rest of the whole protein [15]. In contrast, motifs are short plastic linear sequences with a length of 3 to 15 amino acids. DMIs are the preferential molecular mechanism by which viruses interact with host cells [16]. Motifs are employed by the viruses to mimic and hijack the host cell’s essential process for its survival [17]. Currently, two studies have approached the role of motifs present on essential host proteins for SARS-CoV-2 infection. The research of Mészáros et al. [18] consisted in the prediction of motifs retrieved from Eukaryotic Linear Motif (ELM) resource that were mapped onto the angiotensin-converting enzyme 2 (ACE2) and integrins of the human host. They found conserved motifs on the cytoplasmatic tails of ACE2 and integrin β3 that interacts with several critical regulatory protein domains. This motif information was tested later on experimental binding affinity measurements [19] and found that NHERF3 PDZ1, SHANK1 and SNX27 PDZ domains bind to synthetic peptides of the ACE2, and to the synthetic ATG8 domains, MAP1LC3s and GABARAPs, of integrin β3. Those studies exemplify the utility of motif predictions to guide experimental proposals.

Here contrariwise to the previous researches, we focused on the motifs mapped on the MERS-CoV, SARS-CoV, and SARS-CoV-2 proteomes linked to human protein domains. The frequently matched motifs were compared among the coronaviruses. The motif functionality was inferred through enrichment ontology analysis of its partner domains. The based-motif information obtained could be used as the starting point to develop new therapies to combat these viruses in the future.

## Materials and methods

### Protein sequence retrieval

The SARS-CoV (taxid:694009) and SARS-CoV-2 (taxid:2697049) sequences were retrieved from the NCBI virus repository (accessed on 01 September 2020) [20] using available predefined filters, such as human for host, length of proteins, and the completeness option for sequences. These sequences were firstly filtered based on its report date; then, sequences before 2019 were put on the SARS-CoV dataset. The redundant amino acid sequences were removed with the perl program “fasta_uniqueseq.pl” obtained from FASTA Tool list web page (http://www.ncbi.nlm.nih.gov/CBBresearch/Spouge/html.ncbi/fasta/list.html). The sequences for MERS-CoV were retrieved from the virus variation database [21], using the options as human host, sequence completeness, and collapse for removing redundant sequences. The final number of each viral protein in the datasets ordered by its arrangement on the genome are shown in Table 1. The SARS-CoV protein sequences were grouped together with the SARS-CoV-2 dataset for the analysis due to its small number after eliminating the redundant sequences.

**Table 1:** The total number of non-redundant viral protein sequences for analysis.

Protein	MERS-CoV	SARS-CoV	SARS-CoV-2
ORF1ab	162	4	4003
ORF1a	140		
S	98	5	1135
ORF3a	25	3	421
NS4a	22		
NS4b	36		
NS5	21		
E	6	1	45
M	18	6	125
ORF6		1	73
ORF7a		1	149
ORF8	18	1	146
N	44	1	539
ORF10		1	35
TOTAL	590	24	6671

### Domain-motif data mining process

Our data mining process is based on our previous reported methodology [22], adapted to the data retrieved for the MERS-CoV and SARS-CoV/CoV-2 viruses. It includes three main steps. 1) Literature search. First, we obtained the human genes associated to the SARS-CoV/CoV-2 and MERS-CoV related diseases with pubtator [23]. This tool allows searching in a straightforward manner the reporting genes related to the infections by these viral pathogens in the PubMed literature. These gene names were compared and unified with the information from a recent research published by Perrin-Cocon et al., [11] to form a list of unique gene names. This list was submitted into the UniProt database [24] to obtain the human UniProt IDs that match our query for the next process. 2) Pfam database [25] mining for human protein domains: From the Pfam we downloaded the latest version of the files “Pfam-A.regions.tsv” and “Pfam-A.clans.tsv”. The obtained UniProt IDs that match on the Pfam-A.regions.tsv file were extracted to mine the Pfam-A.clans file. Thereby, it was obtained the Pfam accession, clan ID, Pfam ID, and Pfam description columns that contain information associated with our UniProt ID list. 3) The domain-motif information was mined from the databases of three-Dimensional Interacting Domains (3DID) [26] and ELM [27]. The motif information for 3DID was retrieved from the 3DID-DMI flat 2019 version. From this file, the Pfam IDs, domain-motif name, and the regular expressions (regexp) were extracted and stored in local files which was used as the target file to draw out the information associated with the Pfam IDs previously obtained. In the ELM database, the information came from the files “elm_interaction_domains.tsv” and “elm_classes.tsv”. The first file was the target file to match the Pfam accessions IDs and was then used to take out the domain-motif name, Pfam accession, and the associated regexp from the elm_classes.tsv file. Each regexp was used to match motif amino acid sequences in the protein datasets with the patmatch software [28]. We used linux terminal for each query with the bash command “for ID in `cat file_of_IDs.txt`; do grep $ID target_file.txt; done > extracted_info_file.txt”. The obtained files were also checked manually for concordance with the query IDs.

### Identification of potential functional host-like viral motifs

The potential functional motif identification was based on the percentage of regexp that matches a specific amino acid sequence. To this end, we followed 70% cut-off match as in the previous study [29]. For example, a total of 4003 ORF1ab non-redundant sequences were retrieved for SARS-CoV-2; consequently, a regexp present in more than 70% of ORF1ab proteins signifies that a specific motif matched more than 2802 sequences. Those frequent motifs were also queried on shuffled sequences versions of each protein dataset that was produced with the “shuffleseq” function from the EMBOSS suite programs [30]. If those inferred motifs were found scarcely on the randomized sequences, it reinforces as functional motifs.

### Protein domain enrichment analysis

The protein domain enrichment analysis was carried out with the dgOR package [31] for R statistical language. For this analysis, the Pfam accession numbers were used as input data and the first ten significant (p < 0.05) ontologies based on the hypergeometric test related to gene ontology biological process (GOBP) and Gene ontology molecular function (GOMF) were analyzed.

### Identification of motifs as immune epitopes

The immune epitope database (IEDB) [32] was manually queried for motif sequences with ≥ 5 amino acids, setting the blast parameter of identity more than 70%, and selecting the options “human host”, “all assay types”, and the disease option “COVID-19 and Severe acute respiratory syndrome” as filters. This query analysis was omitted for the MERS-CoV because there is not available information for this pathogen on the IEDB.

### Statistics

The statistics rests on descriptive statistics of the frequent motifs. The obtained information was analyzed by its conjunction and disjunction relationships based on the matching patterns. This analysis was carried out with the help of the web tool for the calculation and drawing of custom Venn diagrams (http://bioinformatics.psb.ugent.be/webtools/Venn/).

## Results

### Literature mining

After removing duplicate gene names among the reviewed publications (data in S1 File), 497 human genes for SARS-CoV/CoV-2 and 65 for MERS-CoV infection were found involved in pathogenesis (Table 2, data in S2 File). The comparison of our mined information with Perrin-Cocon et al [11] showed overlapped gene information (n = 124), and the newly acquired (n = 438), especially for the MERS-CoV viruses. After eliminating the duplicated the rest are theunique gene names (data in S2 File), which were used to search its corresponding UniProt IDs to mine the Pfam, 3DID, and ELM databases for the subsequent regexp match analysis.

**Table 2:** The total number of human gene names obtained from the PubMed literature and compared with Perrin-Cocon et al. [11].

	Present study	Present study ∩ Perrin-Cocon et al.,	Perrin-Cocon et al.,
MERS-CoV	55	10	7
SARS-CoV/CoV2	383	114	352

*∩ means the intersection in the conjunction-disjunction analysis.

### Identification of functional viral protein motifs

The functional regions of proteins are either structured or disordered. However, the proteins of coronaviruses were found mainly ordered according to IUPRED (S1 Fig) [33]. For example, most amino acids of the largest protein ORF1ab and the spike (S) protein were found below the 0.5 score. However, few regions of viral protein were disordered, such as the nucleocapsid (N) protein. In this study, the whole regexp lists obtained from the 3DID and ELM databases (data in S3 File) were mapped on the whole viral protein sequences. The frequent (>70%) regexps that matched amino acid motifs are shown in Table 3 and the data in S4 File.

**Table 3:** Total number of motifs frequently matched by regexp.

	3DID			ELM		
Protein	M-CoV	M-CoV ∩ S-CoV/CoV-2	S-CoV/CoV-2	M-CoV	M-CoV ∩ S-CoV/CoV-2	S-CoV/CoV-2
ORF1ab	65	148	31	8	78	5
S	47	50	38	11	44	12
ORF3a	4	6	24	1	13	28
NS4a	14			25		
NS4b	46			35		
NS5	20			28		
E	19	0	5	6	5	4
M	9	5	15	11	18	9
ORF6			4			18
ORF7a			20			27
ORF8	23	1	14	8	7	15
N	23	27	31	9	27	14
ORF10			2			12
TOTAL	270	237	184	142	192	144

*∩ means the intersection in the conjunction-disjunction analysis.

**Fig 1:**
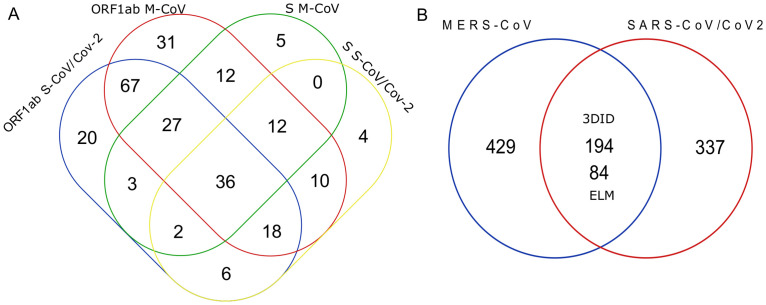
Venn diagrams show the redundant or non-redundant regexp motifs among the proteins or viral proteomes. (A) Venn diagram to show the redundant regexp numbers mapped on the ORF1ab and Spike proteins. (B) Venn diagram of total non-redundant regexp mapped in MERS-CoV and SARS-CoV-2 obtained from the two databases.

The ORF1ab, S, and N sequences were matched by the regexp more than the other proteins from databases. A high number of motifs were shared among three CoVs in the ORF1ab (n = 148 and 78), followed by the S (n = 50 and 44) and the N (n = 27 and 27). The regexp motifs were redundant among the proteins or viral proteomes (data in S4 File); for example, the ORF1ab and S shared the same motifs (Fig 1A); and a high number of motifs shared between the MERS-CoV and SARS-CoV/CoV-2 after removing the redundant (Fig 1B, data in S5 File). Most of these motifs were scarcely on the shuffled sequences; thus, all were considered in the subsequent analysis.

### Protein domain enrichment analysis for non-redundant motifs

**Fig 2:**
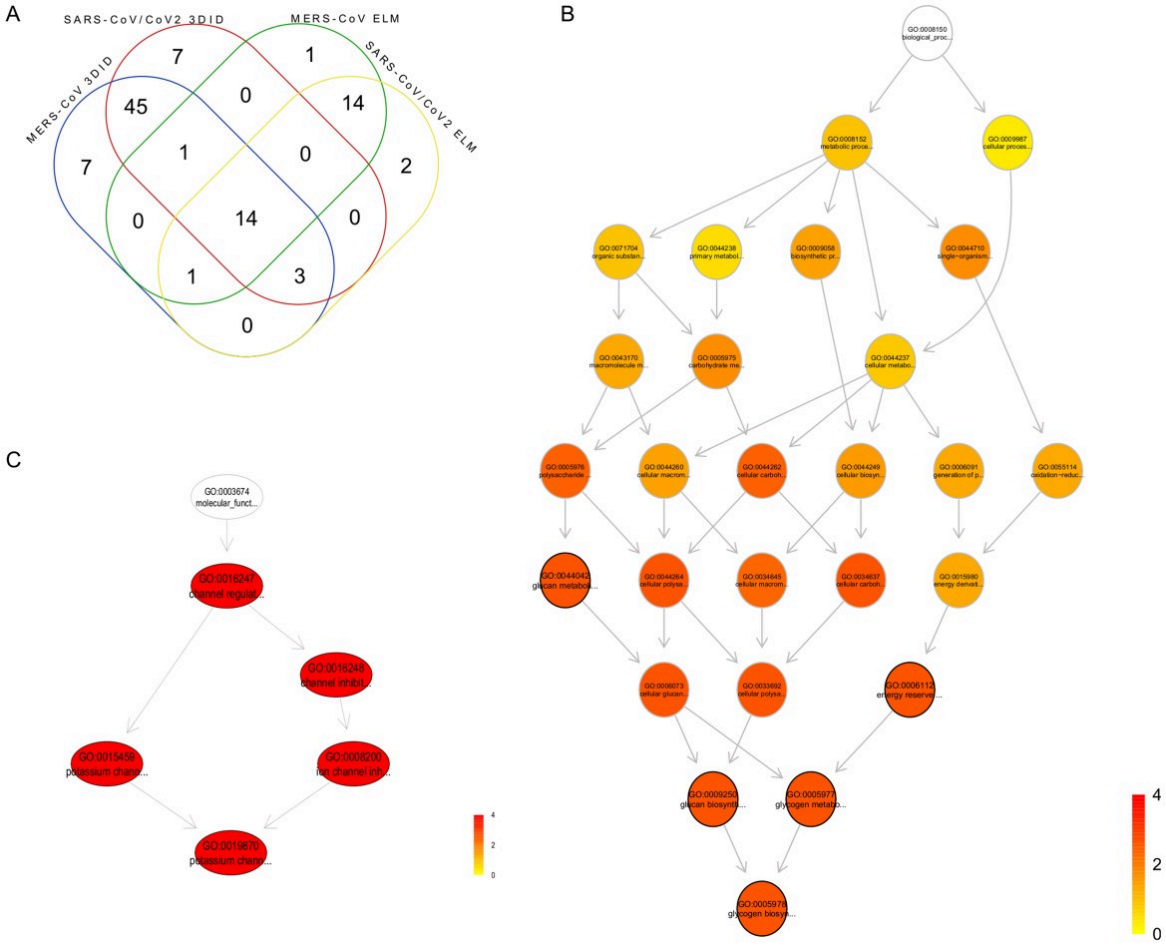
Protein domain enrichment analysis that produced the significant gene ontology terms for non-redundant motifs. (A) Venn diagram for the non-redundant domains. (B) Gene ontology terms for biological processes and (C) molecular functions terms of the non-redundant domains. Nodes are colored according to adjusted p-values.

First, it was examined the conjunction-disjunction relationships for the total number of Pfam accessions associated with non-redundant motifs described above. A total of 78 non-redundant domains were shared for MERS-CoV and SARS-CoV/CoV-2 irrespective of the database source, and few were specific to MERS-CoV (n = 8) and SARS-CoV/CoV-2 (n = 9) (Fig 2A, data in S5 File). Protein domain enrichment analysis of the 78 shared domains for GOBP identifies general terms related to metabolic and cellular processes. Five GOBP significant terms were related to energy reserve and glycogen biosynthesis metabolism (Fig 2B, data in S6 File). GOMF analysis also identifies five important terms related to channel regulation in which potassium channel regulator activity was the most significant (Fig 2C, data in S6 File). The study of specific domains for MERS-CoV and SARS-CoV-2 also showed terms associated with the same biological processes and molecular functions of the 78 shared domains. Thus, those domains could be the primary targets for molecular mimicry generated by MERS-CoV and SARS-CoV/CoV-2 to manipulate the host cell machinery.

### Analysis of significant domains present on distinct host proteins

**Fig 3:**
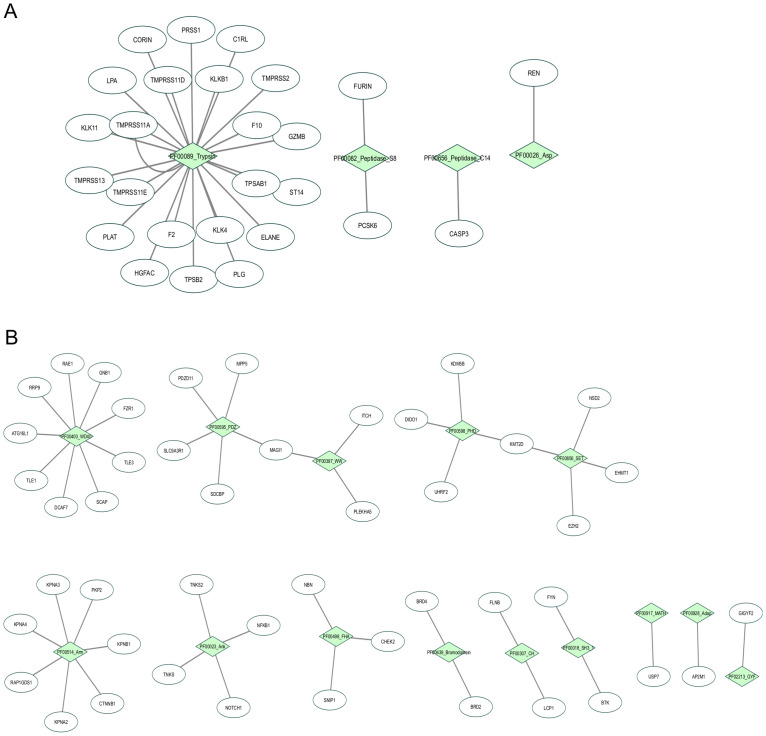
Network representation of significant domains linked to proteins and their gene ontology terms. (A) Biological processes and (B) Molecular functions. The green light diamonds represent the domains, and the ellipses represent the protein names associated with the domains. The images were generated with the cytoscape software [35].

The analysis described above allows us to identify specific proteins linked to the domains involved with significant ontology terms. Four domains (Pfam accession ID: PF00656, PF00026, PF00082, PF00089) related to the glycogen biosynthetic process were present in 26 proteins that matched our gene lists. Among them, the PF00089 related to trypsin domain function is the more promiscuous present on most of the proteins (Fig 3A). This domain was associated with the protease TMPRSS2, an endothelial cell surface protein involved in the entry and spread of CoVs and influenza virus [34], so that this protein has been proposed as a potential drug target to combat those viruses. It was also found the domains associated with the potassium channel regulator activity (Fig 3B).

### Identification of amino acid motif sequences as immune epitopes

**Fig 4:**
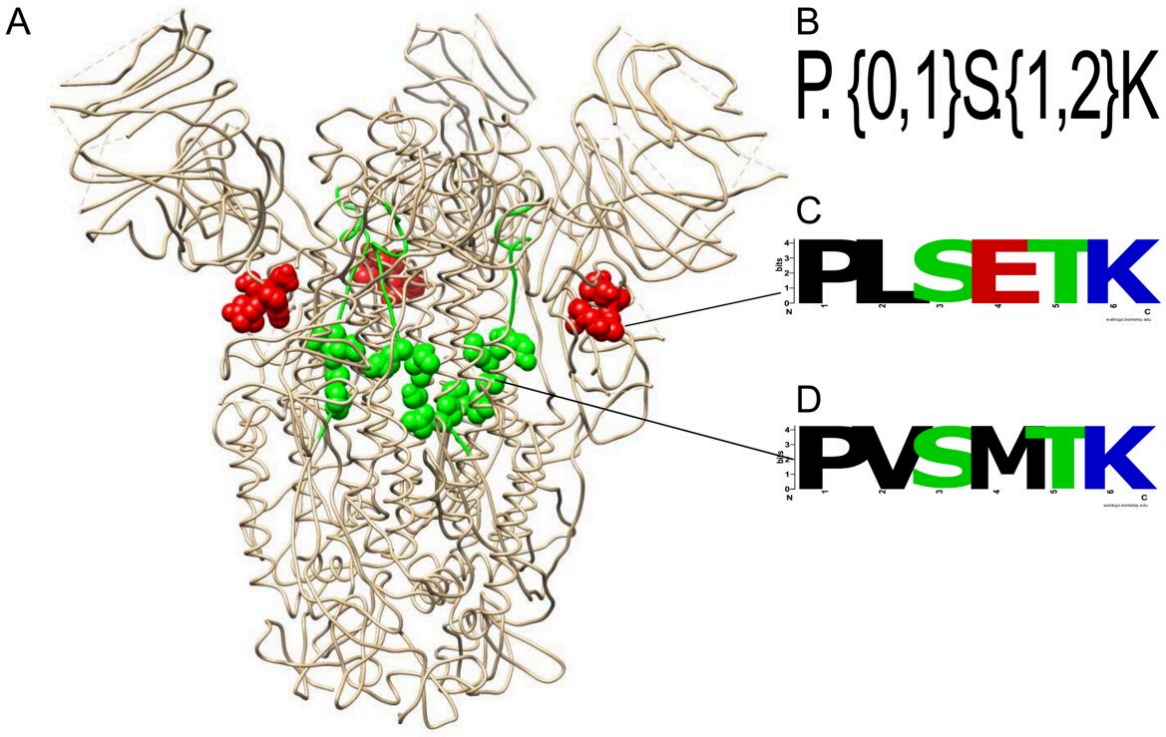
Some motifs matched the epitopes on the spike protein. (A) Spike protein of SARS-CoV-2 (PDBID:6XS6). (B) The regexp. (C) The motif PLSETK. Red balls indicate the PLSETK seqlogo motif mapped at amino acid positions 295 to 300 (D) The motif PVSMTK. Green balls and sticks showed the total length of the epitope ILPVSMTKTSVDCTMYICGD, including the PVSMTK seqlogo motif mapped (the balls) at amino acids positions 728 to 733.

The non-redundant motifs ≥ 5 amino acids were searched for a match with epitopes reported on the IEDB, which were experimentally confirmed. The amino acid sequences of several motifs matched on epitopes sequences for SARS-CoV/CoV-2 that recognize B and T cells specific to class I or II MHC (data in S7 File). These motifs had the following main characteristics. 1) The epitope linear motifs contain the nested motifs recognized by both B and T cells. For example, the motif matched with the regexp [DE]..[IMV].[ST] was found on the B cell and T cell epitope PK**EITVA**TSRTLSYYK (IEDB ID: 48052) in the M protein [36] of SARS-CoV and SARS-CoV-2 [37]. 2) Motifs matched by the same regexp are prone to occur in different protein structural locations. For example, the regexp motif P.{0,1}S.{1,2}K matches the amino acid sequences PLSETK and PVSMTK locating to varying coordinates on the S protein (Fig 4). 3) Motifs maintain its crucial amino acids, and little variations occur at neighbor sites. For example, the **PVSMTK** motif nested on the B cell linear epitope IL**PVSMTKT**SVDCTMYICGD (IEDB ID:1309493) of SARS-CoV-2 (Fig 4A and 4D) [38] varied a little on the epitope sequence **PVSMAK**TSVDCNMYICGDS (IEDB ID: 49968) of the SARS-CoV, maintaining its main amino acid anchors P,S and K. PVSMAK was found only in one SARS-CoV-2 sequence (NCBI ID: QKV39263) isolated from Washington, Yakima County.

## Discussion

In this work, we employed our previous data mining methodology [22] to identify potential functional motifs but applied to MERS-CoV and SARS-CoV/CoV-2 viruses. The main advantage of this method is the search restricted to human protein targets involved in the virus pathogenesis. The initial step allows us to reduce *a priori* the query on the 3DID and ELM databases. As a result, the unsheathed domain-motif information is potentially associated with human genes related to pathogenesis of the MERS-CoV and SARS-CoV/CoV2. Our approach is then similar to the methods used by Hagai, T., et al., Becerra, A. et al and Zhang, A et al [29, 39, 40] in predicting functional motifs. These methods include some distinctive features such as predicting disordered regions on the protein, the high frequency of amino acid motifs in the protein sequences datasets under study, and the scarcity of amino acid motifs on shuffled sequences. The filters were tailored according to the information obtained in each data mining process. All those filtered steps guided our analysis to a more specificity that linked the predicted functional motifs as part of immune epitopes as previously we did for influenza A viruses [22]. It is distinctive of our prediction approach, because it was used to reduce the high rate of false positives associated with the computational prediction of motifs [41]. Furthermore, our method could be an alternative for computer-aided reverse vaccinology.

One interesting result is that the tendency of matched motifs occurred in the most variable proteins, the ORF1ab, and the S protein of the coronavirus proteomes. The ORF1ab contains the nonstructural proteins responsible for the translation machinery of viruses in the intracellular environment [42] and the S protein is essential for the virus’s attachment to the host cell [43]. The tendency of motifs to appear on the proteins involved in virus replication was also observed in influenza viruses [44]. Thus, the high frequency of host-like motifs in those viral proteins suggests that such proteins could be the master kidnappers. Another finding is the high number of shared motifs across the proteome or distinct proteins of a proteome, reflecting the viral motifs to evolve independently in light of acquiring host-like mechanisms for the success in the invasion of host cells.

The domain enrichment analysis showed that the general biological processes, and molecular functions could be the consequence of the MERS-CoV and SARS-CoV/CoV-2 mimicry to hijack the host cell. The most significant ontology terms are the energy-saving and glycogen biosynthesis metabolism association. This result agrees with that viruses use the infected cells’ carbon sources to achieve viral replication and virion production [45]. It is reasonable that glycogen, a storage form of glucose, is utilized in unexpected, exhausting cell activity [46] as infected. On the other hand, as this biosynthetic pathway is vital for the viruses’ survival, targeting essential components such as the glycogen synthase kinase could help treat virus infections. It was reported that the use of two glycogen synthase inhibitors altered the hepatitis C virus assembly and release [47]. Hence, the proteins we found in the present study could be used to explore them as drug targets.

In another context, motifs have been suggested as potential immunogens [41]. It took our attention to search motif that matched with immune epitopes. Indeed we found that some motifs matched to the epitopes on the IEDB. Some of them were nested on the epitopes of earlier SARS-CoV and also present on those new SARS-CoV-2. It reaffirms the evidence of cross-reactive immune responses to coronavirus infections by SARS-CoV and SARS-CoV-2 [48–51]. Additionally, our study identified the epitopes harboring motifs that could interact with human protein domains. It is quite relevant because such domain-motifs shared in the different coronavirus can trigger a common molecular mimicry process that could lead to autoimmune diseases. It was demonstrated that antibodies derived from Flu vaccinated patients react with homologous sequences of the nucleoprotein of influenza A virus and the hypocretin receptor 2 domain of humans, the latter of which was involved in narcolepsy, an autoimmune adverse effect attributed to the Flu-vaccine [52]. Influenza immunization is also attributed to Guillain-Barré syndrome [53], a disease in which its pathogenesis is associated with several bacterial and viral pathogens’ molecular mimicry [54–56]. Thus, our results are vital to helping in the currently underway rational vaccine development efforts, mainly because several autoimmune diseases have been associated with COVID-19 [57].

## Conclusions

In conclusion, this study showed that our method’s adaptability and practicality could guide a rational inference of domain targets and their interacting host-like motifs on the MERS-CoV and SARS-CoV/CoV-2 proteomes. A high number of motifs were shared in the different CoVs, and it could interact with human proteins, indicating that molecular mimicry is a common strategy for CoVs. The finding of motifs as part of immune epitopes makes our method a suitable alternative for reverse vaccinology. The obtained information could be the starting point for future theoretic and experimental studies to develop new drugs and peptidic vaccines to combat those viruses.

## Supporting information

S1 Fig Order and disorder regions for the MERS-CoV, SARS-CoV, and SARS-CoV-2 proteins arranged by its known genome order. (PDF)

S1 File The literature mined information. (XLSX)

S2 File The merged gene name lists for the MERS-CoV and SARS-CoV/CoV-2. (XLSX)

S3 File The regexp lists obtained from 3DID and ELM. (XLSX)

S4 File The redundant regexp matched on the MERS-CoV and SARS-CoV/CoV-2 proteomes. (XLSX)

S5 File The non-redundant motifs with its domain accession partner. (XLSX)

S6 File GOBP and GOMF for the significant domains. (XLSX)

S7 File The motifs nested on linear sequences of epitopes from IEDB. (XLSX)
