# Supplementary material for: The analysis on the human protein domain targets and host-like interacting motifs for the MERS-CoV and SARS-CoV/CoV-2 infers the molecular mimicry of coronavirus
Source: PLoS One. 2021 Feb 17;16(2):e0246901. doi: 10.1371/journal.pone.0246901 (PMC7888644; doi:10.1371/journal.pone.0246901)
Supplement: S1 Fig — (PDF) [file pone.0246901.s001.pdf]

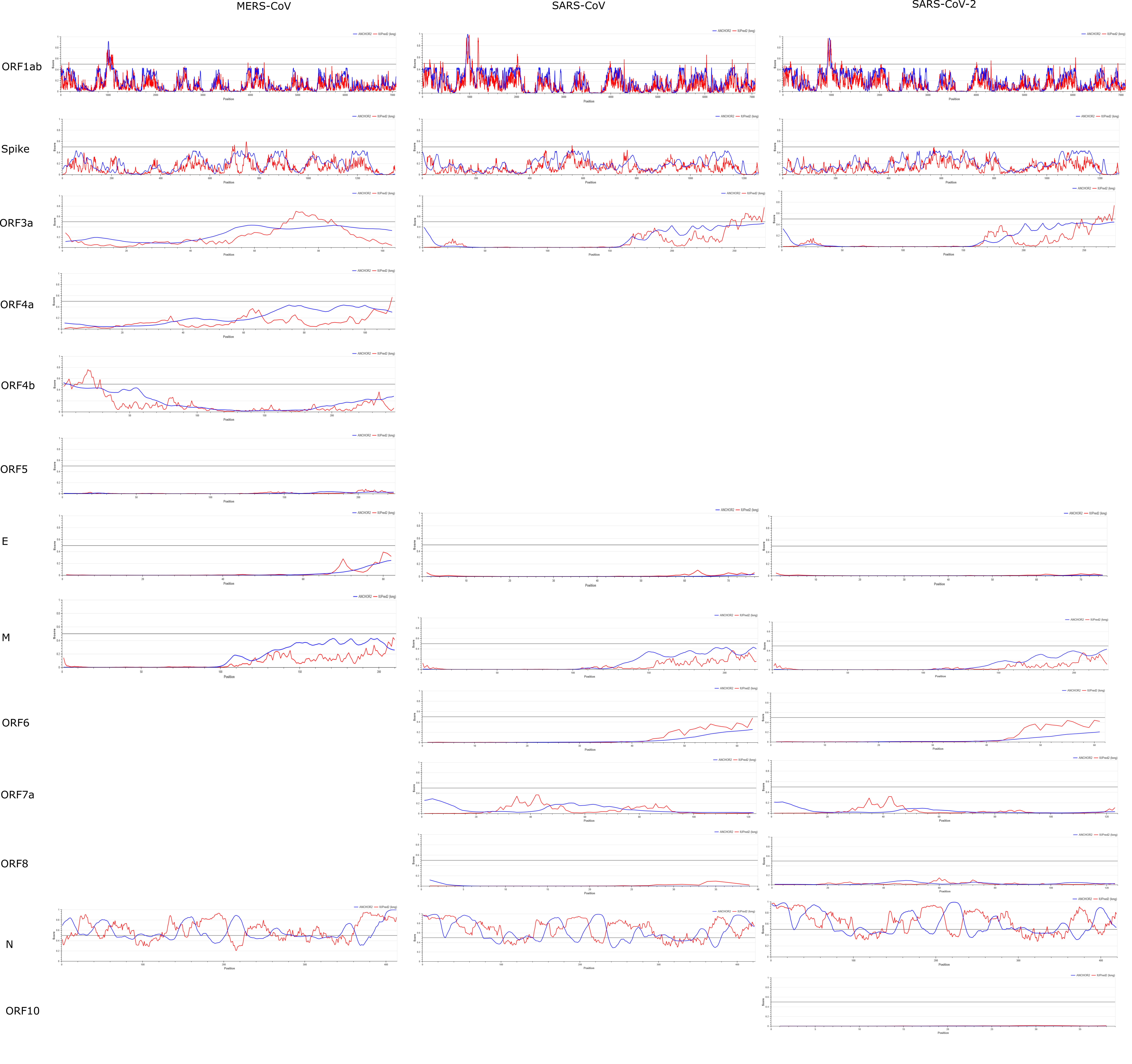

MERS-CoV
SARS-CoV
SARS-CoV-2
ORF1ab
Spike
ORF3a
ORF4a
ORF4b
ORF5
E
M
ORF6
ORF7a
ORF8
N
ORF10
ANCHOR2
IUPred2 (long)
Score
Position
